# The influence of socio-cultural interpretations of pregnancy threats on health-seeking behavior among pregnant women in urban Accra, Ghana

**DOI:** 10.1186/1471-2393-13-211

**Published:** 2013-11-19

**Authors:** Phyllis Dako-Gyeke, Moses Aikins, Richmond Aryeetey, Laura Mccough, Philip Baba Adongo

**Affiliations:** 1 School of Public Health, College of Health Sciences, University of Ghana, Accra, Ghana

**Keywords:** Pregnancy, Perception, Beliefs, Socio-cultural, Healthcare, Ghana

## Abstract

**Background:**

Although antenatal care coverage in Ghana is high, there exist gaps in the continued use of maternity care, especially utilization of skilled assistance during delivery. Many pregnant women seek care from different sources aside the formal health sector. This is due to negative perceptions resulting from poor service quality experiences in health facilities. Moreover, the socio-cultural environment plays a major role for this care-seeking behavior. This paper seeks to examine beliefs, knowledge and perceptions about pregnancy and delivery and care-seeking behavior among pregnant women in urban Accra, Ghana.

**Methods:**

A qualitative study with 6 focus group discussions and 13 in-depth interviews were conducted at Taifa-Kwabenya and Madina sub-districts, Accra. Participants included mothers who had delivered within the past 12 months, pregnant women, community members, religious and community leaders, orthodox and non-orthodox healthcare providers. Interviews and discussions were audio-taped, transcribed and coded into larger themes and categories.

**Results:**

Evidence showed perceived threats, which are often given socio-cultural interpretations, increased women’s anxieties, driving them to seek multiple sources of care. Crucially, care-seeking behavior among pregnant women indicated sequential or concurrent use of biomedical care and other forms of care including herbalists, traditional birth attendants, and spiritual care. Use of multiple sources of care in some cases disrupted continued use of skilled provider care. Furthermore, use of multiple forms of care is encouraged by a perception that facility-based care is useful only for antenatal services and emergencies. It also highlights the belief among some participants that care from multiple sources are complementary to each other.

**Conclusions:**

Socio-cultural interpretations of threats to pregnancy mediate pregnant women’s use of available healthcare services. Efforts to encourage continued use of maternity care, especially skilled birth assistance at delivery, should focus on addressing generally perceived dangers to pregnancy. Also, the attractiveness of facility-based care offers important opportunities for building collaborations between orthodox and alternative care providers with the aim of increasing use of skilled obstetric care. Conventional antenatal care should be packaged to provide psychosocial support that helps women deal with pregnancy-related fear.

## Background

Progress toward Millennium Development Goal 5 has been slow in some resource-limited countries [1]. At the beginning of the Millennium, maternal mortality ratio for sub-Saharan Africa alone was estimated to be nearly 50 times higher than what was reported by industrialized countries [2]. Unfortunately, trends show evidence of little maternal health improvement in sub-Saharan Africa over the last decade [1-6].

In developing countries, evidence suggests direct consequences of pregnancy and childbirth continue to account for most maternal deaths [2]. These outcomes are mainly attributed to haemorrhage, sepsis, and hypertensive complications [2,6]. Structurally, these conditions are seen as outcomes of a complex web of social, economic, educational, political and cultural factors [1,7,8]. Where facilities and appropriate interventions are available, it is estimated 90% of such maternal deaths can be avoided; especially when 15% of these complications develop unexpectedly and become life threatening [1]. Consequently, women are encouraged to receive continuous maternity care from a skilled provider.

In Ghana high rates of maternal mortality remain a public health concern as the 2007 Ghana Maternal Health Survey report estimates a maternal mortality ratio of 580 deaths per 100,000 live births [9]. Due to the high maternal deaths, the Minister of Health declared maternal mortality as a “national emergency” during the 2008 Ghana Annual Health Summit [10]. Research indicates majority (96%) of pregnant women in Ghana received Antenatal Care (ANC) from a trained provider, including, doctor, nurse/midwife or auxiliary midwife; about 77% of these women made four or more antenatal visits during pregnancy as recommended by WHO [9]. However, a skilled attendant is present at approximately half (55%) of all deliveries [9], with 20% and 9% assisted by trained Traditional Birth Attendants (TBA), and untrained Traditional Birth Attendants, respectively. Also, data indicates that nationally, 54% of births are delivered in health facilities, whilst about 45% occur at home [9]. The situation is worse in some parts of the country, like Northern Ghana, where 71% of women are reported to have delivered at home and 25% at a hospital/clinic [11].

The Ghana Maternal Health Survey, 2007 reports that approximately 32% of half of women who did not have a skilled attendant at delivery, claimed it was not necessary to receive skilled attendant at delivery [9]. Worthy of note, is the fact that this large proportion of women who perceive skilled birth attendance as needless, together with women who fail to utilize the service due to identified reasons, all end up delivering without required skilled supervision. This is a grim reality, which warrants an in-depth understanding of the multiple factors that hinder use of facility-based services.

Previous studies indicate structural factors, including lack of financial or economic resources, transportation, and delivery supplies, lack of coordination and referral between TBAs at the community level and facilities can all inhibit women from using facility-based services [9,12-15]. Some studies show that barriers to access, especially financial ones, rather than traditional beliefs, were the main obstacles to delivery at health care facilities [12,16]. However, other studies indicate client’s negative perceptions of healthcare staff, including reports of unfriendliness at delivery also serve as barriers to obtaining skilled care [12-14,16].

Furthermore, researchers emphasize socio-cultural influences on use and non-use of public health facility services in developing countries [17-22]. This includes extensive evidence provided on how gendered social roles and sex differences lead to inequalities in health-related options and outcomes for both women and men [17,23,24]. In some contexts, it has been observed that a woman’s use of antenatal and facility-based delivery services is the outcome of a complex interplay of gendered cultural hierarchies that locate pregnancy-related decision-making in remote authorities such as older female relatives or traditional birth attendants [17,24].

Also, a systematic review which, analyzed determinants of antenatal care use in developing countries, identified cultural beliefs and perceptions about pregnancy as key influential factors [22]. Historically, Ghanaian culture emphasizes pregnancy as a potentially dangerous period that requires spiritual protection [25,26]. Thus, care for pregnant women is multifaceted, involving the medical and also psychosocial, economic and spiritual support. To help address social, cultural and spiritual concerns, the contemporary growth of charismatic and evangelical Christian churches has provided a new avenue for many Ghanaian women to seek protection from the dangers they perceive from the natural and supernatural forces such as witches, wizards, and sorcerers [27]. In this regard, anecdotal reports suggest some women choose to deliver at prayer camps (i.e. residential locations established by various churches, where people can stay for any length of time in order to be healed or have their problems solved through nearness to the benevolent powers that emanate from the presence of a prayer leader) rather than in health facilities [27-29]. Unfortunately, the exact extent of patronage of these camps for delivery is presently unknown [27,28]. Also, Traditional Birth Attendants (TBAs) are noted to enjoy patronage due to their high sensitivity to socio-cultural norms together with a greater ability to incorporate psychosocial care into their services compared to modern health facilities [20,30,31].

Gaps in the continued use of maternity services from a skilled provider in Ghana suggest need for further investigation into the socio-cultural context of provision and utilization of health care services during pregnancy and delivery. Socio-cultural perceptions, which allow or disallow use of pregnancy-related services including psychosocial, medical, and spiritual support, must be examined. Most studies conducted on the influences of socio-cultural norms on use of maternity services in Ghana, have focused on rural communities [12,17,32]. However, current records indicate that urban Ghanaian women do not necessarily deliver at health facilities. Therefore, this study seeks to provide a peri-urban perspective by focusing on the beliefs, perceptions and knowledge about pregnancy and delivery and how they influence care-seeking behavior among pregnant women.

## Methods

### Study design

The qualitative data used for this paper was collected within a larger project that explored pathways by which women accessed pregnancy and delivery care in the Ga East Municipality. The entire study was cross-sectional with survey, cost analysis, and qualitative components. The qualitative part constituted of focus group discussions (FGDs) and in-depth interviews (IDIs). All focus group discussions were conducted as homogeneous groups. FGDs for pregnant women were organized separately based on type of pregnancy care currently used. Women who had delivered within the past 12 months constituted another group, whilst public health care providers constituted a separate group.

### Study area

This study was carried out in the Ga East Municipality located in the north-eastern part of the Greater Accra region. The Municipality is made up of four sub-districts: Madina, Danfa, Taifa and Dome. Ga East area is a mix of urban, peri-urban and rural communities. In 2008, the population was estimated at 294,121, with a growth rate of 4.5%; 76% of the population is urban. The predominant occupations were public service and trading, followed by farming and craftsmanship. The 2008 Annual health Report on the Municipality showed rather low performance on maternal health indicators [33]. For instance, supervised delivery rate was only 38.1% of all deliveries (compared to a national 54%) and antenatal clinic attendance was 69.2% [33]. Both estimates of maternal care were below the national averages.

Two of the four sub-districts, Taifa-Kwabenya and Madina were purposively selected as sites for the data collection. Taifa-Kwabenya was selected because it is a developing community. The limited number of public-managed health facilities in this area, hinders access to health care, including pregnancy and delivery care. Residents in this area seek care from Central Accra, Pokuase, Nsawam, and Amasaman in the adjoining districts. On the other hand, Madina is an old, urbanized and established sub-district with relatively better access to health care as the number of health facilities is more and also has better transport access to Central Accra, and other social services. Most of the health facilities in the Ga East area are located in the Madina Sub-district.

### Research participants

All participants were purposively selected. Participants included mothers who had delivered within the past 12 months, pregnant women, community members, religious and community leaders, professional public health facility worker and individuals who provide alternative maternity services. The researchers first paid several visits to the facilities of pregnancy and delivery care providers, including public health facilities and TBAs. Through these visits the researchers had the opportunity to interact with providers and also observe the delivery and use of pregnancy related services. Pregnant women who were attending antenatal clinics at the time of researchers’ visits were recruited during regular antenatal sessions. Also pregnant women who went to the TBA for antenatal care were recruited during regular sessions. In addition, mothers who have delivered within the past 12 months and also attended Child Welfare Clinics (CWC) with their babies were recruited during regular CWC sessions at the public healthcare facilities. Healthcare providers within the public health facilities (nurses, doctors and midwives) were recruited at the antenatal clinics during regular working hours. Informal pregnancy-related care providers (herbalist, TBA, and Spiritualist) were identified either through health facility workers, who sometimes collaborate with them, or through women who utilize their services.

### Characteristics of study participants

A total of 55 participants were involved in the FGDs and IDIs. These included 35 women; 17 of the women were pregnant during the time of the study, and women who had delivered had their babies between 3 weeks and 12 months before the time of this study (Table 1). The ages of these women varied between 20 and 42 years. A few of the women were unemployed whilst most were engaged in diverse occupations (trading, factory work, healthcare profession and security profession). Several of the women were also married or living with a partner at the time of the study. In addition, 12 health care professionals, spiritualists (3), herbalist (1) and TBA (1) were also involved in the study.

**Table 1:** Study participants

**Focus group discussions and in-depth interviews (N = 55)**					
	**In-depth interviews**		**Focus group discussions**		
**Participants/groups**	**Number of participants**	**Location**	**Number of FGD’s**	**Number of participants**	**Location**
Pregnant TBA clients			1	10	Kwabenya
Pregnant ANC clients			1	7	Madina
Women who delivered in past 12 months	2	Madina-Zongo	1	7	Madina
Women who delivered in past 12 months	1	Ashongman	1	8	Taifa
Nurses/midwives			1	5	Taifa
Nurses/midwives			1	5	Madina
Medical doctors	2	Madina			
Community members	1	Ashongman			
Community members	2	Madina			
Spiritualists	1	Ashongman			
Spiritualists	2	Taifa			
Traditional birth attendant	1	Kwabenya			
Herbalist	1	Taifa			
**Sub-Total**	**13**		**6**	**42**	

### Data collection and analysis

A set of issues was developed to guide the FGDs and IDIs. Key themes covered included perceptions of risk in pregnancy and associated behaviours; medico-religious perceptions; factors related to utilization of care and treatments; use and non-use of health services; health information; issues regarding coverage, utilization and access. Several of the IDIs were conducted in participants’ homes. All of the FGDs and some IDIs were conducted at convenient spaces within public healthcare facilities. Other IDIs were conducted in the shops or homes of the alternative care providers. IDIs and FGDs were conducted in English and in the local Twi language, where necessary. All FGDs and IDIs were audiotaped and transcribed verbatim. Transcriptions were augmented with the researchers’ field notes made during data collection. The data resulting from the transcriptions were evaluated, coded and analyzed by using thematic analysis. Researchers first extracted broad themes and then followed up with coded themes of the text. Themes established considered statements of meaning that were present in most of the data. In an attempt to ensure the credibility of the results codes and themes were corroborated with results from the quantitative and cost analysis components of the study. Emerging themes and categories were used to address the objectives of this study. Ethical approval for this study was obtained from the institutional review board of the Noguchi Memorial Institute for Medical Research, University of Ghana. Also, the researchers obtained written permission from the Municipal Director of Health Services, Ga East, Accra. In addition, verbal consent was received from all participants after explaining the purpose of the study and their right to withdraw their participation at anytime.

## Results

### Beliefs, perceptions and knowledge about threats associated with pregnancy and delivery

Severally, women viewed pregnancy as a biological phenomenon that is shrouded with a lot of uncertainty. Several participants including pregnant women, both ANC clients and TBA clients, women who had delivered and care providers held similar perceptions about pregnancy and delivery. Pregnancy and delivery were widely perceived as critical, “natural,” “painful” and “stressful” experiences that expose a pregnant woman and the fetus to threats. TBA clients, just like ANC clients, identified two types of threats: non-spiritual and spiritual threats. Non-spiritual threats were classified into physical, which includes all forms of general ailments and accidents such as dizziness, fevers, falls and burns. Psychological threats were related to stress emanating from feeling abandoned by their spouses during pregnancy and social threats included lack of social or familial support. Both TBA clients and ANC clients highlighted medical risks such as “lack of medical attention” and “self-medication”. The various quotations below go to support non-spiritual threats to pregnancy identified by respondents:

…your husband is unfaithful and he is somewhere with another woman, you call him and he does not pick, you panic and this can destroy the pregnancy (*Focus group participant, TBA Client, Kwabenya*).

…others too lack support especially if you are living with your husband’s family and they do not like you. You have to force yourself to do things you do not even have to do. Also, the bladder where the baby’s head is can burst when you lift heavy loads (*Focus group participant, ANC attendant, Madina*).

“ …self-medication can destroy the pregnancy, some of the medicines are very harsh and strong, also sugary foods can destroy the blood forming the baby…” (*Focus group participant, TBA Client*).

“…Sometimes if you do not walk well you may fall…” (*Focus group participant, CWC Client, Taifa*)

Most women mentioned that spiritual threats were of concern to them during pregnancy. Pregnant women were perceived to be vulnerable to spiritual attacks that can lead to the destruction of the pregnancy. Many of the participants perceive this spiritual susceptibility as a credible traditional belief. They believe spiritual attacks manifest first in the supernatural and then in the physical. Consequently, miscarriage and other maternity complications can be physical manifestations of such spiritual attacks. A disease condition locally referred to as “a*sram*,” was severally perceived as a physical manifestation of evil spiritual attacks on a pregnant woman. Participants gave varied explanations of “asram” and its manifestations:

“There is a disease in the womb called “*asram*”, it produces heat in the womb and when the baby sleeps in the heat as it develops the baby can die from it , six to seven months latest by eight months the baby will be dead. The stomach will be reducing and…not be moving. But we have our belief that a witch may be responsible” (*In-depth interview participant, Herbalist, Taifa*).

“For me it is about evil eyes and it causes this disease called “*asram*” where some people transfer the disease to the child in the womb or when you eat outside or another person sees the food that you are eating she can do that.” (*Focus group participant, CWC Client, Taifa*)

Some participants believed such spiritual attacks are associated with sorcery or witchcraft, whilst others note the biblical account of God’s curse of women as the rational for this spiritual vulnerability. The comment below given by one community member captures this belief:

“… if you are pregnant you are open to so many things both physical and spiritual and God has already cursed pregnancy from the beginning of creation so even when the person does not take you to a *Juju man* or says it by mouth and if the person has an evil spirit within it will work…when you are pregnant you have to be careful and not fight.” (*In-depth Interview participant, Community member, Madina*)

In addition, participants believed that such spiritual attacks on pregnancy are partially dependent on the specific actions/inactions taken by a pregnant woman. In this context, early disclosure, contact with specific foods/drinks, going out at night, and quarrelsome attitude are actions that can expose a pregnant woman to spiritual attack:

“It is a traditional belief; there are some people that when you tell them and they have evil eyes and mind they can destroy it so unless the pregnancy shows then you tell. If not when it is two or three months you cannot tell.” (*Focus group participant, CWC Client, Taifa*)

… going out in the night one can be met with spirits that may have evil repercussions on you, so you do not have to do that. It can affect you (*Focus group participant, CWC Client, Madina*)

Despite these profound indications of spiritual susceptibility by several women, a few participants who patronized public health facilities discounted some of the traditional beliefs as myths and not practical. For instance one participant questioned the possibility of not going out at night. This claim below particularly shows some challenges in keeping up with traditional beliefs within urban settings:

“For me you can go everywhere. You cannot say you will not go anywhere in the night because you may want to go to church or let’s say you went out and it is late and you have to go home. So for that you cannot say you will not go out at night.” (*Focus group participant, CWC Client, Taifa*)

### Pregnancy-related fear and anxiety

Perceptions of pregnancy as a threatening experience generate fear among several pregnant women. Fear and anxiety is expressed as nightmares, hallucinations, worrying, and sleeplessness. Various women, including both primigravids and multigravids, seeking care from health facilities and alternative care users expressed pregnancy-related fear and anxiety as a common experience:

“I panic when am pregnant. Sometimes I can dream that I did not get the baby after delivery”. (*Focus group participant, CWC client, Taifa*).

“Sometimes you worry a lot, and you hear voices that say ‘you will die’, audibly”. (*Focus group participant, TBA Client, Kwabenya*)

The cause of these fears are either linked to biological conditions, including overdue pregnancy and cesarean section, or knowledge of other women’s negative childbirth experiences. For instance a healthcare professional and other women stated that:

“They (pregnant women) are very much afraid of Cesarean Section so mostly when you (health worker) are referring them they don’t want to go for referral because they know there is a problem.” (*Focus group participant, healthcare professional, Taifa*).

“I fear operation, if you are not able to deliver by yourself. It creates fear in you” (*Focus group participant, CWC Client. Taifa*).

“As for me it is the things people say about it, and the pains that you will go through during delivery, when I remember those things entirely, I get scared” (*Focus group participant, CWC client, Taifa*)

“Some of those who have delivered in the past may scare you that it is difficult” (*Focus group participant, ANC client, Madina*)

Relatedly, some women associate their fears with moments when they are informed about a pregnancy complication and also how healthcare professionals announce that condition to them. This sentiment was widely expressed by TBA clients, and few antenatal clinic clients:

“Sometimes they scare you at the hospital; they can even say that they cannot see the baby ”(*Focus group participant, TBA Client, Kwabenya*).

“At the antenatal sometimes the way they will break news to you will make you panic and even when you are eating you live in fear and panic” (*Focus group participant, CWC client, Taifa*)

Despite these elaborate indications of pregnancy-related fear, few participants who received facility-based antenatal care during their current pregnancy said they could overcome fear through regular antenatal attendance and also assurance from previous positive maternity experiences. For instance, one participant said: “ I was going for my antenatal regularly and was going back to them (healthcare workers) with questions so there was nothing like fear” (*Focus group participant, CWC Client, Madina*).

### Care-seeking behavior

Evidence from the study revealed that pregnant women receive pregnancy-related care from multiple sources based on the nature and type of threats they associate with their pregnancy. Pregnant women utilized both public and private formal health facilities as well as non-orthodox facilities such herbalists, spiritualists/prayer camps and TBAs. Both antenatal clinic and TBA clients seek biomedical care during the initial stages and endorsed it as ideal for safety purposes and emergencies:

“For safety, is good to deliver in the hospital. At times you will deliver the child and the mother may not be healthy so they would have to be rushed to the hospital, you may be bleeding at home but the hospital you can be protected from all these” (*Focus group participant, CWC client, Taifa*)

Nonetheless, participants were quick to highlight experiences with facility-based care that lead pregnant women to either change professional healthcare provider or seek care from a TBA. Providers’ impatience, long waiting time, insufficient time with provider and unfriendly attitude of staff were noted as key reasons why some participants would change providers:

“Some get discouraged when the nurses do not give them the “proper care” that they need, so they stop coming” (*Focus group participant, ANC client, Madina*)

“I first delivered at Legon hospital but I did not like their attitude so I delivered my second child at Ridge Hospital and later because of distance I went to Achimota because it was close to me.” (*Focus group participant, CWC Client, Madina*)

“For some of the pregnant women when you talk to them like that and tell them about a complication, if there is any TBA around they rather go to that place, rather than the health facility they have been referred to” (*Focus group participant, healthcare provider, Taifa*).

### Non-orthodox therapies

Pregnancy is perceived as a potentially dangerous period requiring diverse forms of support, which includes spiritual, psychosocial and medicinal. First, the need for spiritual protection was widely expressed by care providers as well as pregnant women. It was also evident that seeking non-orthodox treatment is common among conventional and non-conventional antenatal clinic attendees and it is usually sourced from pastors, spiritualists and TBA’s. Kinds of spiritual support ranged from a simple act of personal prayer through attending regular prayer sessions to participation in specially organized prayer camps. These spiritual acts spanned the entire pregnancy period, beginning from conception until delivery. The following statements indicate these kinds of spiritual support:

“With pregnancy you need medical attention which is physical and you also have to go before God because a doctor cannot do everything. They can perform an operation on someone but at the end the person may die. But because we know that all things are worked by God if you involve prayers also it helps a lot.” (*In-depth interview participant, spiritualist, Taifa Burkina*)

“We do not joke with pregnancy when it comes to prayers and all that, you can pray on even a hospital bed. It is not everything that you have to go and stay in the prayer camp, you can go but you have to come back” (*In-depth interview participant, Community Leader, Madina*)

Secondly, pregnant women seek psychosocial support to help manage pregnancy-related fear and anxiety. Psychosocial support is mainly sourced from TBAs and spiritualists in this setting. Health care providers (nurses and medical doctors) acknowledged pregnant women seek psychosocial support from non-conventional providers particularly when they are experiencing maternity complications and fear:

“it’s all because of fear and the fact that they have the belief that they (pastors/spiritualist)can do something about their situation because even any time you switch on the TV , Pastors are talking about the fact that if you are to be operated upon and you think that you don’t want to be operated they can pray for you and it will be changed and if you are a pregnant woman and you don’t want to be operated upon they can pray for you and at times you will even deliver in the prayer camps. So at times it all depends upon the fear and all those things” (*In-depth interview participant, medical doctor, Madina*).

Also TBAs were known to provide such psychosocial support through pampering and also with the involvement of other relatives of the pregnant woman. Both the TBA and her clients agreed to the relevance of psychosocial support during pregnancy. The TBA described pregnancy care as being “all about pampering.” Akin to her description, a TBA client confirms that she receives a comprehensive care from the TBA compared to conventional antenatal:

“She (TBA) pampers us as compared to the Nurses, Doctors and Midwives at the hospital. She is patient with us and has time for us. If you are not able to deliver then she takes you to the hospital. She will even involve your husband in the whole process.” (*Focus group participant, TBA Client, Kwabenya*).

The third non-orthodox treatment is herbal therapy usually sourced from herbalists, TBAs, and some spiritualists. Unlike spiritual and psychosocial support, use/non-use of herbal therapies is related to participants’ perception of efficacy, as well as participants’ use/non-use of conventional antenatal services. Mostly, conventional antenatal clinic clients remained committed to biomedical care and were not likely to use any herbal therapy. Yet, few antenatal clinic attendees admit to combining herbal therapy with prescribed orthodox medication and “alternate” between them for a complementary effect that will better manage maternity complications. Others also resort to it because prescribed medications could not treat their condition:

“At the initial stages of my pregnancy I was bleeding and I came to the hospital for drugs but it was persistent. So I went for herbal medicine and it helped me” (*Focus group participant, ANC client, Madina*)

Comparatively, high patronage of herbal therapy was reported among non-conventional antenatal attendees (TBA clients) than among conventional antenatal clinic clients. TBA clients widely endorsed the usefulness of herbal therapy against the perceived negative effects of orthodox medication:

“It (herbal medicine) makes the baby strong and healthy and you don’t experience pain as compared to the hospital drugs” (*Focus group participant, TBA Client, Kwabenya*)

It (herbal medicine) gives strength, the hospital drug weakens me when I take them but the traditional medicines make me active (*Focus group participant, TBA Client, Kwabenya*)

Even though herbal therapy is used during the entire pregnancy period, providers confirmed availability of wide range of herbs used for different pregnancy needs and conditions such as malaria, “*abode*” (fibroid), vomiting, bleeding, infertility, etc. To a large extent, providers of herbal medicine believed in the complementary role of herbal therapies and orthodox medicine. The Spiritualist believes that “*aduro boa aduro*,” literally meaning, treatment regimes rely on one another for effectiveness. The practitioners of non-orthodox medicine allow their patients to take advantage of the plural medical system that exist in the urban environment. For example, a TBA claimed she allows her clients to visit the hospital for medical attention, whilst giving them herbal therapies at particular stages of the pregnancy. This is indicated by the quotation below:

“Some women go to the hospital, but some feel they also need traditional medicine so they come here. They go to the hospital and also take the herbal medicines. We have the one that will cure diseases in the stomach and we the one that make the pregnancy period to be smooth and the one to open women when the time is due.”(*In-depth interview participant, TBA, Kwabenya*)

### Seeking non-orthodox care

Unique patterns in non-orthodox care-seeking were evident in accounts from TBA clients and alternative pregnancy care providers including herbalist, TBA, and spiritualist. TBA clients indicated their decision to seek or not to seek conventional antenatal care can be equally influenced by them, their relatives or friends, or providers of such care. In this case the TBA claims she requests clients to also get registered at the public health facility. Some TBA clients go to the TBA first and only go to a health facility with the TBAs permission. However, a few TBA clients state they will not go to the hospital due to previous unpleasant experiences with facility-based care.

“I came to Auntie first and she told me to go to the hospital, but me I will never go to the hospital if am pregnant again, if Mama (TBA) tells me to go there I will not go” (*Focus group participant, TBA Client, Kwabenya*)

Evidence from alternative care providers (herbalist, spiritualist and TBA) confirmed this referral pattern. They refer clients to healthcare facilities due to their (herbalist/spiritualist) inability to assist in delivery and lack of adequate human resources for emergency cases (TBA). Alternative care providers mainly describe their services as being complementary to facility-based care and not substitutive. The herbalist, spiritualist and TBA invariably draw differences between their skill, and that of health facility-based professionals, acknowledging their inability to offer medical care:

“I tell them when they come that I do not assist in delivery so they should go to the hospital and register so that when they are due for delivery, they know where they are going.” (*In-depth interview participant, Herbalist, Taifa*)

“I encourage them to go to the hospital; they do not have to force themselves to come here just because I take care of them. Everything ends at the hospital, so they have to go there, so you do not put your hope on me while I may not be around, so you have to go there…”(*In-depth interview participant, TBA, Kwabenya*)

“…when you come here … we make you go to the hospital and when there is a problem, hospital is important…” (*In-depth interview participant, Spiritualist, Anyemma Gonno*)

## Discussion

This study examined beliefs, knowledge and perceptions about pregnancy and delivery, and care-seeking behavior among pregnant women in Ga East Municipality. Evidence from this study shows care-seeking behavior of pregnant women is largely mediated by socio-cultural influences that shape individual perceptions of threats to pregnancy. There is a distinct interplay between cultural beliefs about pregnancy, women’s expression of fear, and the ways they seek and use available maternity care services. Women expressed fear of caesarian section, possible loss of baby, labour pains, and other maternity complications. Although previous studies found women exhibiting similar fears about their baby’s wellbeing, childbirth, and hospitalization [19,34,35], this study shows fears may be augmented by the wide range of socio-cultural beliefs about threats associated with early disclosure, sorcery, and witchcraft. Indication of these beliefs shows, not only their ability to thrive within urban settings, but also lead to actions that may disrupt continued use of facility-based maternity care; a behaviour identified amongst both antenatal and TBA clients in this study, albeit differently. It was observed that antenatal clients expressed similar cultural beliefs and fears, therefore combined use of facility-based services and alternative services during pregnancy, but severally delivered at the health facility. On the contrary, TBA clients who expressed similar cultural beliefs and fears, combined use of facility-based services and alternative services, but delivered with help of the TBA. Additionally, women who had delivered using public health facility were likely to resist some cultural beliefs and associated fears.

The disruptive role of socio-cultural perceptions in the use of public health facility services among pregnant women has been identified in other contexts [17-19,21]. In rural Zimbabwe, it was observed that a local belief about increased vulnerability to witchcraft during early pregnancy period resulted in avoidance of health facilities, as against increased use of alternative forms of care [18]. Also, researchers found a high cultural value attached to home births, which sharply contrast with negative perceptions that equated skilled birth attendance with loss of status, loss of control over delivery process, or loss of secrecy during delivery, in Northern Ghana [17]. Similarly, some Tanzanian women are known to hold the belief that long labor may be caused by extramarital affairs during pregnancy, therefore, some avoid delivery at the health facility due to fear of being exposed [19].

Relatedly, the evidence highlights an overwhelming belief in religio-cultural dimension of threats. This notion does not only suggests crucial interplay between the physical and spiritual, but also the need to seek both medical and spiritual therapies [27]. Women severally explained maternity complications as physical manifestations of spiritual attacks leading to the use of faith healing as a health delivery option [27]. Consequently, most pregnant women including regular antenatal clinic clients and TBA clients claim they seek spiritual support from diverse sources to help address these perceived dangers. In Ghana, the high prominence of African syncretic and Pentecostal churches, continues to provide a faith healing avenue where pregnant women can express faith in a Superior Power that can alleviate their health problems [27,32]. Usually, faith healing requires specific actions, some of which might not necessarily encourage use of health facilities [18,27].

Faith healing actions range from offering personal prayers to a Supreme Being, to camping at residential prayer locations where there is constant access to a prayer leader who possess powers believed to offer protection for the pregnancy. However, actions undertaken relate not only to socio-cultural influences, but also experiences of pregnancy complications. In this case a pregnant woman is likely to solicit the help of a faith healer if orthodox medicine is unable to address her condition. In Zimbabwe, faith healers are believed to possess powers that enabled them to protect the woman and the pregnancy from harm, thus they played a key role in the care of pregnant women during the early stages [18]. Also, in rural Ghana, a study indicates relevance of religion on use of maternal health services [32]. For some pregnant women faith healing practices may work to reduce the tendency to use available public health care [32].

In addition, the findings extensively suggest pregnant women’s need for psychosocial and emotional support also shapes the ways they utilize available maternity care services. However, mechanisms through which such needs inform choice of care might be distinctly different for conventional and non-conventional users of antenatal care. Unlike conventional antenatal care users, TBA clients consistently compared public health facility services with alternative services and often made choices considering previous unpleasant experiences with health facilities, as well as their emotional needs. Although, public health facilities are available in this urban setting some women prefer the TBA’s services, because she can better address their psychosocial needs. When fear becomes paramount, a TBA’s ability to provide strong psychological and emotional support during and following delivery becomes a better option [8,20,31] as opposed to pregnant women’s expressed skepticism of public health facility-based care [17,31].

Studies in several rural communities confirm women’s preference for TBAs provision of psychosocial support, and culturally sensitive services, in addition to being caring, accessible, available and trustworthy [17,30,31]. These experiences are different from reports of healthcare workers’ negative attitudes and insensitivity, and women’s lack of confidence in healthcare staff, which discourages use of public health facilities [16,22], which is described by participants as not receiving “proper care”. Beyond confirming this established difference in services, our study also suggests that availability of public health facilities within urban settings do not necessarily ensure use. On the contrary, utilization may be based on expressed need and health delivery options perceived to better address those needs.

Moreover, the health-seeking behavior of pregnant women suggests sequential or concurrent use of facility-based care and alternative forms of care, including herbalists, spiritualists/prayer camps and TBAs. Often faith healers focus on the spiritual, while the public health facilities attend to the physical. Also, some pregnant women use a range of herbal therapies provided by TBAs, herbalists and Spiritualists. However, observations indicate slight differences in reasons for use of herbal medicines, for instance. Several TBA clients used herbal medicines regardless of the condition of their pregnancy, expressing the belief that it strengthens the baby. On the contrary, a few ANC clients who claimed to use herbal medicines indicated this was due to pregnancy complications that orthodox medication could not treat. It is worthy to note that both users and providers of non-orthodox therapies believed in its complementary effect (“*aduro boa aduro*”).

Although, pluralistic care-seeking behaviour has been previously identified among pregnant women [8,36], it is worthy to note that this study also confirms the high patronage of public health antenatal services within urban settings. Reports from developing countries show high utilization of antenatal clinic services, often determined by socio-demographic, structural, cost, contextual, as well as cultural factors [6,11,22,37-40]. Unfortunately, high utilization of antenatal care services in many developing countries has not translated into use of skilled attendant at delivery [6,11,30,41]. A United Nations report indicates 65% of deliveries in developing countries are attended by a skilled health personal, compared to only 46% in sub-Saharan Africa [6]. In Ghana, the Maternal Health Survey shows 96% of pregnant women received antenatal care from a skilled provider. However, this percentage decreases dramatically for skilled assistance at delivery and postnatal care following delivery (55%) [9]. The situation was worse in the Northern part of the country where a recent study detected as high as 71% home deliveries [11]. Also a recent study in Ethiopia found as high as 71% of women received antenatal care from a health professional, yet only 16% of deliveries were assisted by health professionals [30].

High antenatal care use vis a vis low skilled attendant at delivery could partially be explained by observed patterns in the concurrent and sequential use of multiple sources of care. Our findings indicate possible influence from non-orthodox service providers who usually initiate and encourage use of multiple forms of care. This was particularly the case for TBA clients; highlighting gendered cultural hierarchies that locate pregnancy-related decision-making in remote authorities other than the pregnant woman [24]. In this study some TBA clients claimed decisions regarding use of pregnancy-related care are influenced by the TBA and the pregnant woman’s husband. Decisions may be deferred to the TBA because she is seen as an authority on traditional pregnancy-related practices [42]. This is similar to findings from a study conducted in rural Ghana, which indicate older female relatives and traditional birth attendants along with the woman’s husband, decide if and when the laboring woman should seek care [17]. This situation grants the pregnant woman limited control over decisions made regarding use of facility-based care. However, it is worthy to note that in practice, gender roles and relations are frequently negotiated and produces fluid patterns of behaviour depending on space, time and status [24]. Consequently, not all pregnant women will be equally influenced by known gendered norms.

More importantly, we note that use of combination of therapies deepens the contradiction where facility-based care is privileged for antenatal care and emergencies, but not necessarily deliveries [19]. Other studies observe situations where women attended antenatal clinics in order to acquire antenatal attendance card needed to ensure care in case of complications later [19]. This is due to an unfortunate perception that pregnancy is a natural process and only warrants medical attention when there are complications [16,43].

Regardless, skilled obstetric care during delivery has been identified as one of the most important ways to address maternal mortality, and in sub-Saharan Africa, this is often achieved by encouraging deliveries in healthcare facilities [44,45]. It is assumed that a skilled health professional can administer interventions to prevent and manage life-threatening complications, such as heavy bleeding, or refer the patient to a higher level of care when needed [6]. Estimates show that use of skilled obstetric care at delivery could reduce maternal mortality by 13–33% globally [45]. In this light, the Ghana Health service initiated MDG Acceleration Framework (MAF) program, which seeks to prioritize skilled delivery [10],must be customized to address key socio-cultural beliefs about pregnancy. Also, attempts should be made to investigate the extent to which any kind of fear driven by cultural beliefs discourages pregnant women from using skilled obstetric care at delivery.

To ensure the appropriate use of maternity care, women must be educated to appreciate facility-based maternity care as a total package. Pregnant women need to access antenatal care, which facilitates the detection and treatment of problems during pregnancy and provides opportunity to inform women, and their families about their health and danger signs associated with pregnancy [9]. Traditionally, children are delivered at home in Ghana with the assistance of birth attendants or elderly women in the community. However, an important component of efforts to reduce health risks of mothers and children is to increase the proportion of babies delivered under medical supervision [9,45]. Proper medical attention can reduce risk of infection and increase timeliness of effective intervention [9,45]. Similarly, postnatal care is useful for detecting and monitoring potential complications that if unattended, could result in the death of the mother and or her baby.

In this study we interviewed mothers who had delivered within the past 12 months, pregnant women, community members, religious and community leaders, formal and informal healthcare providers to highlight perceived physical and spiritual threats that drive certain care-seeking behaviour. A limitation, however, is that the results of the qualitative data based on a small purposively selected sample may not be representative of the entire population of pregnant women in the Ga East area. Despite these limitations, it is remarkable to note that this study provides important insights on perceived threats that drive multiple care-seeking practices among pregnant women within urban Accra. Moreover, the use of qualitative techniques allowed us to gain a better understanding on beliefs, knowledge and perception on pregnancy and delivery, and was used to triangulate findings generated by the cost analysis and the survey components of the larger project.

## Conclusions

Socio-cultural perceptions regarding threats to pregnancy shape how pregnant women seek available pregnancy-related healthcare services. Perceived physical and spiritual threats drive commonly expressed pregnancy-related fear among pregnant women and their use of conventional healthcare, together with alternative psychosocial, spiritual and herbal therapies. Although, facility-based care is privileged over other forms of care, care-seeking patterns indicate psychological needs may disrupt continued use of health facility care throughout the pregnancy-delivery continuum. We would argue that such distractions may be profound among women whose use of antenatal care was initiated by alternative care providers; in which case the alternative providers wield some control in decisions taken. Based upon these conclusions, we recommend the following:

•Development of a comprehensive maternity care model that encourages continued use of facility-based care starting from pre-pregnancy through pregnancy, delivery, and beyond. Services must address psychological needs of pregnant women, by encouraging healthcare professionals to interact, Identify and provide for those who require psycho-education.

•Despite gaps in utilization patterns, facility-based care is viewed positively. This offers important opportunities to encourage women to deliver with a skilled attendant in a health facility. Therefore, efforts should be made programmatically to emphasize need for skilled birth attendance in addition to accessing antenatal care.

•The possibility that alternative care providers can initiate facility-based care visits by pregnant women offers a unique opportunity for strengthening collaboration among conventional and non-conventional care providers. In this regard, a unique “referral” system that offers recognition and or a reward to alternative care providers who initiate use of facility-based care, must be developed.

•The commonly pluralistic care-seeking pattern among pregnant women suggests the need for research among HIV-positive pregnant women. We need to explore HIV-positive pregnant women’s healthcare seeking patterns to know if they also use multiple forms of care, and also the extent of risk involved in seeking alternative forms of care whilst being on antiretroviral therapy.

## Competing interests

The authors declare that they have no competing interests.

## Authors’ contributions

PDG participated in data collection and qualitative data analysis of the study. PBA participated in the design of the study. MA participated in the design, undertook the cost analysis and wrote the initial manuscript of the study. RA helped in the initial proposal development and coordinated the data collection and management and data analysis. LM participated in the design of the study. All authors were involved in drafting, revising the manuscript, reading and approving the final manuscript.

## Pre-publication history

The pre-publication history for this paper can be accessed here:

http://www.biomedcentral.com/1471-2393/13/211/prepub
